# Prevalence of Myopia and Its Associated Risk Factors Among Children Attending a Tertiary Hospital in Saudi Arabia

**DOI:** 10.7759/cureus.37045

**Published:** 2023-04-02

**Authors:** Hashem S Almarzouki, Suzan Y Alharbi, Dohaa A Bakhsh, Sarah N Alayoubi, Nada O Taher, Fayssal Farahat

**Affiliations:** 1 Department of Ophthalmology, King Abdulaziz Medical City, Ministry of National Guard - Health Affairs, Jeddah, SAU; 2 College of Medicine, King Saud Bin Abdulaziz University for Health Sciences, Jeddah, SAU; 3 Research Center, King Abdullah International Medical Research Center, Jeddah, SAU; 4 College of Medicine, King Saud bin Abdulaziz University for Health Sciences, Jeddah, SAU; 5 Research Center, King Abdullah International Medical Research Center, Riyadh, SAU; 6 Department of Infection Prevention and Control, King Abdulaziz Medical City, Ministry of National Guard - Health Affairs, Riyadh, SAU; 7 College of Public Health and Health Informatics, King Saud Bin Abdulaziz University for Health Sciences, Riyadh, SAU

**Keywords:** pediatrics myopia, tertiary center, shortsightedness, refractive errors, myopia

## Abstract

Clinical Relevance: This study serves as a tool for optometrists and ophthalmologists to reinforce adherence to several preventive factors, which may decrease the incidence of myopia, and avoidance of risk factors by multiple means including education during hospital visits. It also provides insight into who should be screened and tailored screening programs for children.

Background: While studies assessing the prevalence of myopia in Saudi Arabia have contradicting results, only a few studies have evaluated the risk factors of myopia and the effect of using electronic devices on its occurrence. Thus, this study aimed to determine the prevalence of myopia and associated risk factors, among children attending an ophthalmology clinic in King Abdulaziz Medical City, Jeddah, Saudi Arabia.

Methods: A cross-sectional study was conducted. A total of 182 patients aged < 14 years were selected using convenient sampling. Direct assessment of the refraction was performed in the clinic, and a questionnaire was completed by the child's parent.

Results: Of 182 patients who met the inclusion criteria, 40.7% had myopia. Myopia was more common in boys (56.8%) than in girls (43.2%), with a median age of 8.7 years. Using multivariate regression analysis, only age (eight years and above) (OR=2.15, CI=1.12-4.12, P= 0.03), and family history of myopia (OR= 5.83, CI= 2.82-12.05, P= 0.001) were significant predictors of myopia in children. Other variables (e.g., sex, and use of laptops, computers, smartphones/tablets, or TV) were not statistically significant.

Conclusions: This study did not show a statistically significant association between using electronic devices and the onset and progression of myopia among children. Studies with a larger sample size are required to further investigate this association and assess other potential risk factors.

## Introduction

Myopia is one of the most common refractive errors with an estimated 22.9% prevalence among the general population worldwide [1,2]. Patients with myopia can clearly see objects in close proximity. However, distant objects appear blurred and are barely seen. This is caused by the incompatibility between the axial length of the eye and the power of optical elements [1,3]. A recent epidemiological study conducted in various geographical areas showed that the prevalence of myopia has significantly increased globally over the past half-decade and will continue to increase. Additionally, it is predicted that by 2050, 49.8% of the general population worldwide will suffer from myopia, which is a concern as, if not corrected, myopia can have short- and long-term consequences on the quality of life [2]. Patients with high myopia tend to have reduced quality of life impacting their productivity, mobility, and everyday life due to several adverse influences from psychological, financial, and cosmetic factors resulting from wearing thick spectacle lenses [4].

Recently, most cross-sectional studies have reported variations in the prevalence of myopia among school-aged children from different geographic areas and ethnic backgrounds [5]. For example, the estimates of myopia in 12- and 17-year-old school children of Asian ethnicities in Australia were reported to be 42.7% and 59.1%, respectively, whereas the estimates of myopia in European Caucasian children of the same ages were 8.3% and 17.7%, respectively [5]. Recent investigations of myopia concluded that both genetic and environmental factors play an important role in the development of this condition [5]. Many studies have been carried out to investigate various environmental variables that may have a role in the development of myopia. The results on the effect of near-work activities, such as reading or using hand-held technology, have been contradicting [6-10]. On the other hand, numerous studies have revealed that spending time outside has a detrimental and protective effect on myopia [8,10]. Moreover, studies have shown that parental myopia is not only a risk factor for having myopia, but it is also a risk factor for progressive myopia in offspring [11-14].

A few studies have been conducted in Saudi Arabia to identify the prevalence of refractive errors in school-aged children [6-8]. Among those studies were cross-sectional studies performed in primary schools in the Al-Qassim and Alhassa regions, which estimated an overall prevalence of 16.3% and 13.7% of correctable visual impairment, respectively [6,7]. Furthermore, a study performed on school-entrant children at King Abdulaziz Medical City in Riyadh demonstrated an estimated refractive error of 4.5%, in which myopia ranked the highest with a prevalence of 2.5% [8]. Another study conducted in Medina among children attending the pediatrics ophthalmology clinic estimated the prevalence of myopia to be approximately 3.5% [9]. Although a few studies have been conducted in Saudi Arabia to determine the prevalence of refractive errors among school-aged children, local studies that have addressed myopia as an entity on its own including its risk and preventive factors are extremely scarce [6-8].

A recent meta-analysis involving 30 randomized controlled trials (RCTs) evaluated different interventions that can reduce the progression of myopia, including pharmacological agents such as atropine and pirenzepine. Moreover, additional spectacle lenses have also been proven to be effective in significantly reducing its progression. Guggenheim et al. found that time spent outdoors was predictive of myopia incidence [10]. Another meta-analysis documented that each increase in hours spent outdoors per week was associated with a 2% reduced odds of myopia [8]. This is supported by the light hypothesis, in which it is speculated that light simulates dopamine to antagonize the development and progression of myopia [11].

Identifying the risk factors associated with myopia as well as accurate measurement of its prevalence is crucial in order to suggest some preventive measures or implement educational campaigns that aim to increase awareness of the importance of outdoor activities and exposure to the sun. Thus, our study aimed to identify the prevalence of myopia among children up to 14 years of age who are attending the outpatient ophthalmology clinic in King Abdulaziz Medical City, Jeddah, Saudi Arabia. We also aimed to investigate the associated risk factors and provide insight into the preventive measures against myopia in our population.

## Materials and methods

This cross-sectional study was conducted at the outpatient ophthalmology clinic in King Abdulaziz Medical City, Jeddah, western Saudi Arabia between September 2016 and December 2019. Inclusion criteria were patients aged < 14 years who had attended the clinic and were diagnosed with a refractive error. Patients with ocular diseases other than refractive errors were excluded. A convenient sampling technique was used to select study participants.

Patients were included based on a documented clinical diagnosis of myopia or non-myopic disorder. Direct assessment of refraction has been performed in the clinic by pediatric ophthalmology specialists. Furthermore, every child underwent a complete ophthalmic examination by a qualified optometrist, including slit lamp examinations and ophthalmoscopes. For patients under the age of four years, a cycloplegic refraction was performed by installing two drops of 1% cyclopentolate, spaced five minutes apart, into each eye individually, followed by a retinoscopy. For children older than 10 years, subjective refraction was carried out as long as auto-refraction readings were consistent. Young children's visual acuity was evaluated using an Allen picture chart, whereas older age groups were evaluated using a Snellen eye chart.

The data collection sheet was collected from the children’s guardians. The questionnaire was validated for face and content validity by two independent experts. A pilot study was conducted to measure questionnaire internal consistency using Cronbach’s alpha. The dependent variable was myopia diagnosed by a pediatric ophthalmology specialist. Independent variables included age, gender, presence of any disease before myopia, positive family history of myopia, electronic device usage (e.g., laptops, smartphones, computers, iPad (Apple Inc., Cupertino, California, United States)/tablets, TV, etc.), average duration spent on electronic devices daily, time spent on reading books, and under-corrected eye problems. Other independent variables were daytime sun exposure and duration spent on outdoor activities.

Ethical consideration

This study was approved by the Institutional Review Board (IRB) of King Abdullah International Medical Research Center, Jeddah, Saudi Arabia (approval number: SP17/306/J). Informed consent forms were obtained from the children’s guardians before their participation in the study.

Statistical analysis

The obtained information from the data collection sheet was entered into Microsoft Excel 2007 (Microsoft Corporation, Redmond, Washington, United States). After that, data analysis was performed using IBM SPSS Statistics for Windows, Version 22.0 (Released 2013; IBM Corp., Armonk, New York, United States). Descriptive statistics (e.g., mean and median, percentage, and frequency) were reported. Chi-square test was performed for categorical variables (myopia (yes, no), gender (male, female)). Student’s t-test was used to compare the means of the two group variables (myopia [yes, no] and age in years). Additionally, logistic regression analysis with a 95% confidence interval (CI) was applied to identify the associated risk factors associated with the occurrence of myopia. The level of significance was determined at p < 0.05.

## Results

The current study included 182 pediatric patients whose ages ranged from newborn to 14 years. Of the 182 participants, 92 (50.5%) were males and 90 (49.5%) were females. Overall, myopia prevalence was 40.7% with a 95%CI (33.5%, 48.2%). Myopia was seen in more males (56.8%) than females (43.2%) (p=0.17). The prevalence of myopia in children aged ≤ 8 years was 47.3%, which increased to 52.7% in those aged > 8 years, this difference was statistically significant (p=0.03). The median age of patients with myopia was 8.7 years, whereas non-myopic participants had an average age of 7.1 years. In terms of independent variables related to myopia, of the total participants, 37 (20.3%) had other diseases before the diagnosis of myopia, with strabismus being the most common (2.2%). Fifty (27.5%) participants had a family history of myopia diagnosed at an early age, mostly in first-degree relatives (19.8%) (Table 1).

**Table 1:** Univariate analysis of variables associated with occurrence of myopia among the studied children. *Chi square test

Variables	Myopia		p-value*
	Yes (n=74) n (%)	No (n=108) n (%)	
Gender			
Male (n=92, 50.5%)	42 (56.8)	50 (46.3)	0.17
Female (90, 49.5%)	32 (43.2)	58 (53.7)	
Age category			
≤8 years (n=104, 57.1%)	35 (47.3)	69 (63.9)	0.03
>8 years (n=78, 42.9%)	39 (52.7)	39 (36.1)	
Family history of myopia (n=50, 27.5%)	35 (47.3)	15 (13.9)	0.001
Use of electronic devices			
TV (n=129, 70.9%)	55 (74.3)	74 (68.5)	0.40
Smartphones (n=122, 67.0%)	49 (66.2)	73 (67.6)	0.85
Tablets (n=64, 35.2%)	30 (40.5)	34 (31.5)	0.21
Laptops (n=27, 14.8%)	15 (20.3)	12 (11.1)	0.09
Desktop computers (15, 8.2%)	9 (12.2)	6 (5.6)	0.11

Among patients with myopia compared to those with non-myopic disorders, 74.3% used TV (P=0.40), 66.2% used smartphones (p=0.85), 40.5% used tablets (p=0.21), 20.3% were using laptops (p=0.09), and 12.2% used desktop computers (p=0.11). The estimated average time in minutes spent daily on different devices was higher in the myopic group compared to the non-myopic group; however, none of these differences was statistically significant (p>0.05). Among the myopic patients, mean (SD) time in minutes spent daily on TV, smartphones, tablets, laptops, and desktop computers was 108.85 (131.1), 90.81 (138.30), 47.35 (87.73), 20.95 (65.63), and 13.75 (42.71), respectively.

Using logistic regression analysis, among several factors that were assessed for the risk of developing myopia (age, sex, family history of myopia, and use of TV, smartphones, tablets, laptops, and desktop computers), only ages above eight years (OR=2.15, 95%CI=1.12-4.12, p=0.03) and positive family history (OR= 5.83, 95%CI= 2.82-12.05, p=0.001) were the significant predictors. Use of TV (OR= 1.32, CI= 0.61-2.84, p=0.48), smartphones (OR= 0.62, CI= 0.30-1.27, p=0.19), tablets (OR= 1.42, CI= 0.70-2.91, p=0.33), laptops (OR= 2.01, CI= 0.83-4.90, p=0.12), and desktop computers (OR= 1.65, CI= 0.48-5.67, p=0.43) did not show statistically significant association. The overall model was statistically significant (p= 0.001), Nagelkerke R2= 0.25, and model overall classification percentage was 72.5% (Table 2). 

**Table 2:** Multiple logistic regression analysis of factors associated with occurrence of myopia among the studied children.

Variables	Odds Ratio (OR)	95% Confidence Interval	p-value
Gender (males vs. females)	0.65	0.33, 1.28	0.21
Age category: >8 years vs. ≤8 years	2.15	1.12, 4.12	0.02
Family history of myopia	5.83	2.82, 12.05	0.001
Use of TV	1.32	0.61, 2.84	0.48
Use of smartphones	0.62	0.30, 1.27	0.19
Use of tablets	1.42	0.70, 2.91	0.33
Use of laptops	2.01	0.83, 4.90	0.12
Use of desktop computers	1.65	0.48, 5.67	0.43

## Discussion

The current study reported the prevalence of myopia among children less than 14 years old who attended an outpatient ophthalmology clinic in western Saudi Arabia. The prevalence of myopia was identified as 40.7%, which is almost similar to findings of another study among Asian school children aged 12 years (42.7%), although it was much lower than data from the same study, where the prevalence of myopia reached 69% among older children 15 years of age [12]. On the contrary, the current findings were far higher than data reported among European Caucasian children of the same age (8.3%) and African ethnicity (5.5%) at 15 years of age [1]. Variations in the prevalence of myopia in different studies may be related to the study design, whether hospital-based versus community-based data. However, variations according to ethnic background and geographical region have been reported in several studies. East and Southeast Asian countries were found to have the highest prevalence [12]. Comparatively, European and Australian children have lower rates of myopia, and African people also have the lowest prevalence [13,15]. This can be supported by the fact that myopia is a complex condition involving several genes, and its increased prevalence among first-degree relatives or specific ethnicities is explained by the shared genetic makeup as well as other potential epigenetic factors, such as environmental and lifestyle factors [16].

In comparison to national studies, the prevalence of myopia in our study is much higher than that in primary school children in Qassim, Saudi Arabia (5.8%) and among children attending pediatric ophthalmology clinics in Al-Madinah (3.5%) [2,9]. Moreover, it is more prevalent than in a study conducted on kindergarten and primary school entrants in Riyadh (2.5%) [3]. However, the current prevalence is lower than that reported in another national study conducted on primary school children aged 6-14 years in different areas of Al-Hassa (65.7%) [5].

Myopia was reported in more than half of the males in our study (42 out of 74 myopic participants), which contradicts a study conducted on Caucasian children in Poland in which more females were affected (431 out of 5865); this might be due to the larger sample size used in the Polish study (5865 children) in comparison to our sample size (182) [6]. At a national level, according to a study conducted at pediatric outpatient clinics in Al-Madina, similar to our study, myopia was more prevalent in male than female participants [9]. Additionally, a previous systematic review by Tang et al. reported an increased risk of exotropia with myopia [17].

Although the use of certain electronic devices, such as televisions, tablets, laptops, and computers showed increased risk, the difference was not statistically significant. The systematic review by Wang et al. suggested a link between smartphone overuse and ocular symptoms, including myopia, particularly in children [18]. Another systematic review and meta-analysis of 27 articles found that near-work activities (e.g., watching TV, reading, playing video games, etc.) increase the risk of developing high-degree myopia. For this, the authors recommended controlling time spent on near-work activities to reduce the risk of developing myopia among children [19]. A previous school-based study reported by Liu et al. revealed a higher association of myopia with time spent on computers, but not with time spent watching TV [20]. Furthermore, our study reported that the presence of a family member diagnosed with myopia at an early age, especially first-degree relatives, was associated with an increased risk of myopia. This finding correlates with a study reported by Liang et al., which also found strong familial effects on the development of myopia even after the adjustment of environmental factors [21]. Lastly, other epidemiological studies suggested greater time spent outdoors might be associated with reduced prevalence of myopia [7,8]. To the best of our knowledge, exposure to electronics and outdoor activities has not been studied at the national level, which hinders a local comparison.

The findings of the current study may be limited because of the cross-sectional design, convenient sampling technique, relatively small sample size, and being a hospital-based conducted on children who complained of refractive errors in a tertiary care hospital. These limitations may affect the generalizability of information; however, the current study highlighted the increased prevalence of myopia among children and raised several questions related to behavioral and lifestyle risk factors associated with the occurrence of myopia at a very young age.

## Conclusions

Age and family history are predictors of myopia according to the findings of this study. The use of electronic devices did not show a statistically significant association with the occurrence of myopia, which may be related to the study's small sample size. Multi-centered studies should be conducted to further assess the use of electronic devices and the impact of outdoor activities and sun exposure on the reduction of the incidence of myopia among children. Initiating an early screening program for children with a positive family history of myopia is also recommended, in addition to conducting awareness campaigns that educate parents and schoolteachers about symptoms of refractive errors including myopia among children.
